# Partial Geometric Frustration in Inorganic Supramolecular Spin Systems with One-Dimensional Trigonally Aligned Magnetic Chains _∞_^1^(MCl_4_)^2−^ (M = Fe^2+^, Co^2+^)

**DOI:** 10.1038/srep17344

**Published:** 2015-12-09

**Authors:** Xiao-Ming Jiang, Xiao-Guo Li, Ming-Jian Zhang, Zhi-Fa Liu, Yong Liu, Jun-Ming Liu, Guo-Cong Guo

**Affiliations:** 1 Institute for Quantum Materials, Hubei Polytechnic University, Huangshi 435003, P. R. China; 2 State Key Laboratory of Structural Chemistry, Fujian Institute of Research on the Structure of Matter, Chinese Academy of Sciences, Fuzhou, Fujian 350002, P. R. China; 3 Laboratory of Solid State Microstructures, Nanjing University, Nanjing 210093, P. R. China

## Abstract

Exploration of new spin systems with low-dimensional subunits have been of great interest in the past decades due to their interesting physical properties and potential applications in molecular spintronics. Two inorganic supramolecular complexes, (Hg_3_S_2_)(FeCl_4_) and (Hg_3_S_2_)(CoCl_4_), with trigonally aligned 1-D infinite magnetic _∞_^1^(FeCl_4_)^2−^ or _∞_^1^(CoCl_4_)^2−^ chains have been prepared by solid-state reactions. They exhibit 3-D long-range spin order with strong field dependence and field induced metamagnetic behavior. The intrachain and interchain magnetic coupling constants were estimated by DFT+*U* and DFT+*U*+SOC calculations and the both complexes can be regarded as partially frustrated spin systems. The spin Hamiltonian was constructed, the ground state is proposed to be incommensurate spiral spin order, which differs from the commensurate 120° spin structure ground state of fully frustrated trigonal case by a little canted angle. This study shows that cooperative magnetic ordering induced by geometric frustration can be realized in inorganic supramolecular systems assembled by weak van der Waals’ interactions.

Complexes with discrete one-dimensional (1-D) magnetic subunits have attracted much attention due to their high uniaxial magnetic anisotropy and quantum tunneling relaxation, which may find applications in the areas of information storage and molecular spintronics^1^. Different from many organic and inorganic-organic hybrid single chain magnets, in which organic ligands and supramolecular interactions (hydrogen bonding^2^, π−π stacking^3^, etc.) are often adopted to create 1-D magnetic chains, inorganic supramolecules are generally the aggregates of different covalent-type hosts and guests assembled by weak noncovalent interactions like electrostatic and van der Waals’ interactions^4^, and have the advantages of simultaneous introduction of semiconductive subunits. The combination of semiconductive and magnetic subunits within one single molecule should be an effective way to discovery multifunctional molecule-based magnetic materials, especially for the noncentrosymmetric ones, in which nonlinear optical^5^, ferroelectric^6^, and multiferroic properties^7^, may be exhibited.

HgQ (Q = S, Se, Te) materials are very important II-VI semiconductors and well known for their high carrier mobilities, good electric and optical properties. They are widely used as IR detectors, LED’s and switches^8^. The 3-D diamond-type structures of HgQ can be flexibly truncated by dimension reduction agents^9^, of chlorides of metals like In, Bi, Cu, Ag, Zr, Hf and so on, to form diverse 3-D mixed structures^10^,^11^, 2-D layers^12^,^13^,^14^, and 1-D bands^15^, supporting the opportunities to obtain low-dimensional systems, especially the single-chain and single-molecular magnetic chalcohalides. For the 2-D HgQ layers with hexagonal honeycomb voids, geometric spin frustration can be easily formed if these voids are filled with antiferromagnetically coupled subunits. Geometric spin frustration is a type of spin configurations that can constraint a large fraction of magnetic sites to randomly adopt several nearly degenerate spin configurations resulting in disordered ground states^16^. Representative geometrically frustrated magnetic lattices include antiferromagnetically coupled 2-D Kagome lattices, triangular and hexagonal lattices, and 3-D tetrahedral lattices^17^. Magnetic ordering phenomena in (partially) geometrically frustrated spin systems are subject of numerous studies, and it can lead to spiral spin order and *E*-type antimagnetic order in some multiferroics exhibiting multifunctional ferroelectricity and magnetism^7^.

The metal chalcogenide hosts in supramolecular metal chalcohalides like (Hg_3_S_2_)(Bi_2_Cl_8_)^13^ and (Hg_3_Te_2_)(UCl_6_)^14^ not only stabilize low-dimensional metal halide guests chemically, but also can modulate the magnetic coupling strengths between different magnetic guests, which play a key role in forming special spin order and exhibiting interesting magnetic properties. Although lots of metal chalcohalides have been found^18^,^19^,^20^, rare work on magnetic assemblies has been reported in such systems, except MnPnQ_2_X (Pn = Sb, Bi; Q = S, Se; X = Cl, Br, I)^21^, Ce_53_Fe_12_S_90_×_3_ (X = Cl, Br, I)^22^, Ba_4_Fe_2_I_5_S_4_^23^, and (Hg_3_Te_2_)(UCl_6_)^13^.

During the exploring supramolecular transition metal chalcohalides with low-dimensional magnetic subunits, we synthesized two new isostructural supramolecular complexes (Hg_3_S_2_)(MCl_4_) (M = Fe^2+^ (**1**), Co^2+^ (**2**)), whose crystal structures feature 1-D infinite _∞_^1^(MCl_4_)^3−^ chains perpendicularly penetrating the 2-D (Hg_3_S_2_)^2+^ host layers. This structural feature is strikingly different from the reported chalcohalides based on HgQ^12^,^13^,^14^, where all the 0-D guests are embedded between the (Hg_3_Q_2_)^2+^ layers.

Complexes **1** and **2** exhibit 3-D long-range ordering induced by antiferromagnetically coupled interchain partially lifted geometric frustration, resulting in the field induced metamagnetic phase transitions. To the best of our knowledge, the both complexes are the first examples exhibiting cooperative magnetic ordering induced by geometric frustration in inorganic supramolecular systems. Herein, we report their crystal structures, magnetic properties and theoretical explanation based on first-principles calculations.

## Results and Discussion

### Synthesis & new phases determination

The single crystals of both complexes were obtained by oxygen-free mediate-temperature solid-state reactions and the product yields are of about 80% and 95% for **1** and **2**, respectively. Pure crystals with clear and polyhedral shape of **1** and **2** for physical property measurements were handpicked under microscope and their purities were confirmed by Powder XRD study (Fig. S1). The both complexes are stable in air and water.

The structures of **1** and **2** were determined by single crystal X-ray diffraction and their formulas are solved based on taking collectively into account crystallographically refined compositions and requirements of charge neutrality. Relevant crystallographic data and details of the experimental condition for **1** and **2** are summarized in Table 1. Atomic coordinates and selected interatomic distances are reported in Tables S1 and S2 in the Supporting Information.

The thermogravimertric analysis (TGA, Fig. S3) indicate that both **1** and **2** can be stable up to about 300 °C. Semi quantitative microscope analysis of single crystals using energy-dispersive X-ray spectroscopy (EDS) confirmed the presence of Hg, Fe, S, and Cl in the approximate molar ratio 3.0:0.8:2.0:3.6 for **1**, and Hg, Co, S and Cl in the approximate molar ratio 3.0:0.8:1.8:4.1 for **2**, and no other elements were detected, which are in agreement with stoichiometric ratio of formula of both complexes determined from X-Ray diffraction. The diffuse reflectance spectra of **1** and **2** reveal the presence of optical gaps of 1.93 and 1.64 eV (Fig. S4), respectively, which are consistent with their red (**1**) and blue (**2**) colors.

### Crystal Structures

**Figure 1:**
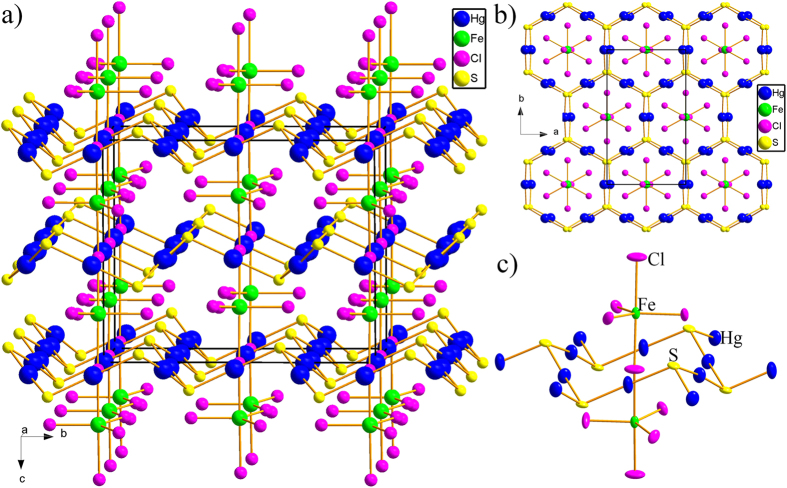
View of structural framework of **1** along the *a* direction (a) and the *c* direction (b), and coordination geometry of **1** (c). Complex **2** is isostructural to **1** and its structure can be obtained by replacing Fe^2+^ positions in **1** with Co^2+^.

The structures of **1** and **2** are isostructural, they are characterized by 2-D (Hg_3_S_2_)^2+^ polycationic host layer and 1-D infinite chiral chain (FeCl_4_)^2−^ in **1** and (CoCl_4_)^3−^ in **2** (Fig. 1). The (Hg_3_S_2_)^2+^ layer is consist of edge-linked Hg_6_S_6_ rings in the chair conformation with linearly coordinated Hg^2+^ cations and trigonal pyramidal coordinated S^2−^ anions. The guests in both complexes are built of trigonal bipyramidly coordinated Fe^2+^ or Co^2+^ cations to form FeCl_5_ or CoCl_5_ trigonal bipyramid, which apex-shared with each other to form a 1-D infinite _∞_^1^(FeCl_4_)^2−^ or _∞_^1^(CoCl_4_)^2−^ chiral chain with a 2_1_ screw axis along the *c* direction. It is interesting to find that the 1-D guest chains penetrate perpendicularly the 2-D (Hg_3_S_2_)^2+^ host layers through hexagonal voids, the structural feature is significantly different from all the reported typical metal chalcohalides containing similar Hg_3_Q_2_ (Q = S, Te, As) host layers, such as (Hg_3_SAs)(GaCl_4_) (**3**)^24^, (Hg_3_Q_2_)(Bi_2_Cl_8_) (Q = S (**4**), Te)^13^ and (Hg_3_Te_2_)(UCl_6_) (**5**)^14^, where all the discrete guest polyanions (GaCl_4_)^−^, (Bi_2_Cl_8_)^2−^ and (UCl_6_)^2−^ are embedded between the host layers.

**Figure 2:**
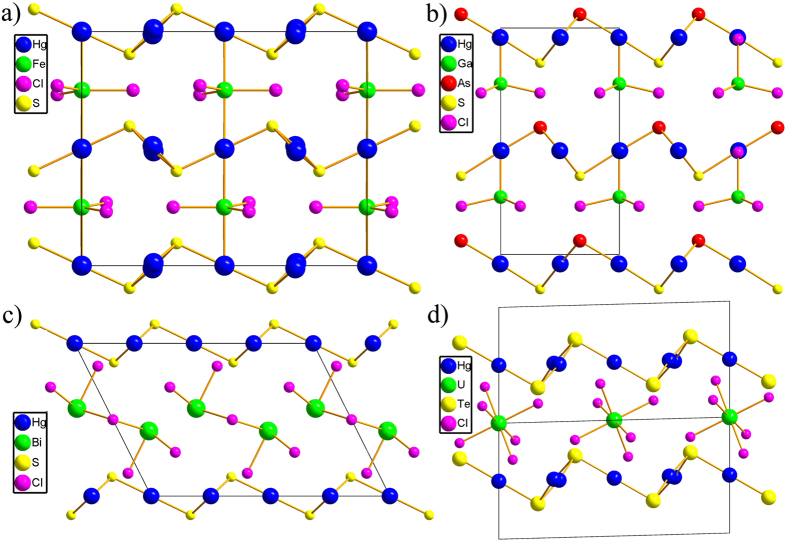
View of different relative locations of neighboring (Hg_3_Q_2_)^2+^ (Q = S, Te, As) layers in (Hg_3_S_2_)(FeCl_4_) (a), (Hg_3_SAs)(GaCl_4_) (b), (Hg_3_Q_2_)(Bi_2_Cl_8_) (c) and (Hg_3_Te_2_)(UCl_6_) (d).

**Figure 3:**
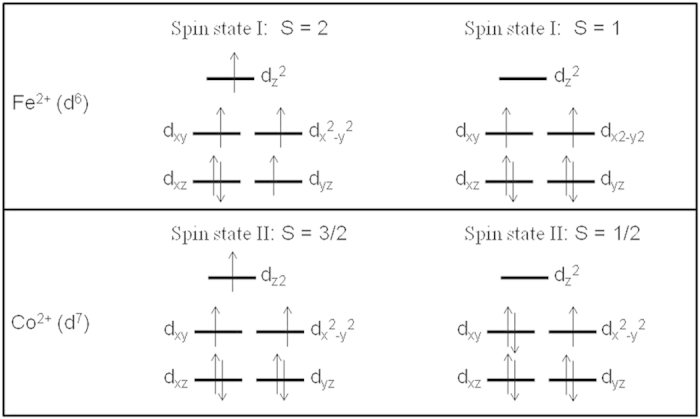
Possible ground spin states of Fe^2+^ and Co^2+^ in **1** and **2**.

A comparison can be made among these five structures (Fig. 2), the relative locations of neighboring Hg_3_Q_2_ layers can be categorized into two types, complexes **1** and **2** (Fig. 2a) and **3** (Fig. 2b) belong to type I, in which the neighboring Hg_3_Q_2_ layers are antiparallel with a mirror symmetry in the middle of them, and complexes **4** (Fig. 2c) and **5** (Fig. 2d) belong to type II where the neighboring Hg_3_Q_2_ layers are parallel with some horizontal shift in **4** and no shift in **5**. The relative locations of host Hg_3_Q_2_ layers depend on the dimension, symmetry, size and charge of guest polyanions. The size of void between Hg_3_Q_2_ host layers can be modulated by parallel/antiparallel arrangement and horizontal shift to fit and stabilize smaller mononuclear polyanions like (UCl_6_)^2−^ in **5** and larger binuclear polyanions (Bi_2_Cl_8_)^2−^ in **4**. Due to the charge balance of the whole structure and charge +2 of Hg_3_Q_2_ unit, the discrete 0-D polyanions normally posses charge −2, except the case in **3** where one half of S^2−^ are replaced by As^3−^, then the charge of Hg_3_Q_2_ unit is decrease to +1 of Hg_3_SAs unit and it can stabilize polyanion GaCl_4_ of charge −1. For complex **1** and **2**, although Fe^2+^ and Co^2+^ is five-coordinated, the FeCl_5_ or CoCl_5_ units can apex-shared with each other to decrease the average charge of the polyanion to −2, so Hg_3_Q_2_ layers can also stabilized 1-D guests in addition to the reported 0-D polyanions. The comparison among these metal chalcohalides with similar structure features indicate Hg_3_Q_2_ chalcogenide layers exhibit high flexibility to stabilize different kinds of guest polyanions, some other metal chalcohalides may be predicted based on the commonly adopted coordination types of some metal cations and location relationships between them and Hg_3_Q_2_ layers.

The Hg–S bond lengths in the cationic layers of **1** and **2** range from 2.347 (3) to 2.360 (4) Å, which lie in the normal ranges for Hg–S bond lengths in known mercury chalcohalides^12^,^14^, and Fe–Cl bond distances (2.274(5)–2.629(1) Å) and Co–Cl bond distances (2.217(6)–2.669(1) Å) in the anionic guest chains of **1** and **2** are close to those found in the relevant metal chlorides. The Fe–Cl bonds (~2.63 Å) and Co–Cl bonds (~2.67 Å) along the *c* direction are much longer than those Fe–Cl bonds (~2.27 Å) and Co–Cl bonds (~2.22 Å) in the *ab* plane, and are very close to the estimated dividing lengths (~2.66 Å for Fe–Cl bond and ~2.65 Å for Co–Cl bond) between covalent bonding and Van der Waals interactions. However, the Fe–Cl and Co–Cl bonds along the *c* direction in both compounds can be still regarded as covalent bonding and make contribution to the crystal field splitting of *d* orbitals of Fe^2+^ and Co^2+^, due to the fact that the intrachain Fe−Fe and Co−Co magnetic interactions are much stronger than interchain magnetic interactions based on the first principles calculations to be presented below.

The distances between the cationic layers and anionic chains in **1** and **2** are significantly longer than the expected values for covalent bonding, thus suggesting the typical host-guest supramolecular interactions between them. The shortest interatomic distances between the halogen atoms of the guest chains and the mercury atoms in the host layers of **1** and **2** are 3.080 (1) and 3.218 (4) Å, respectively, which are much longer than the Hg–Cl covalent bond length (~2.48 Å) but shorter than the sum (~3.51 Å) of the Van der Waals radii of mercury and chlorine. This indicates the weak Van der Waals interactions between the cationic and anionic moieties in the structures of **1** and **2**, as the case found in other inorganic supramolecular complexes^25^,^26^.

### Magnetic properties

The Fe^2+^ (*d*^6^) and Co^2+^ (*d*^7^) ions in both complexes are trigonal bipyramidly coordinated by five Cl^−^, resulting in the *d* orbital degeneracy lifting to three energy levels, (*d*_xz_, *d*_yz_), (*d*_xy_, *d*_x_^2^_−y_^2^) and *d*_z_^2^ (Fig. 3). Both Fe^2+^ and Co^2+^ exist two possible spin states, high spin *S* = 2 and low spin *S* = 1 for Fe^2+^ and high spin *S* = 3/2 and low spin *S* = 1/2 for Co^2+^. The Fe−Cl−Fe and Co−Cl−Co angles in both complexes are close to 180^o^, and the intrachain nearest neighbouring Fe−Fe and Co−Co pairs should exhibit antiferromagnetic interactions. Although the interchain magnetic coupling interactions should be weak because of the long distances (~7.5 Å in **1** and **2**) between the neighbouring chains, they may play a predominant role in forming long range magnetic order, and can also result in spin frustration due to the very close trigonal arrangement of the spin sites in the **b**plane (Fig. 1b) if antiferromagnetic coupling exists between them.

**Figure 4:**
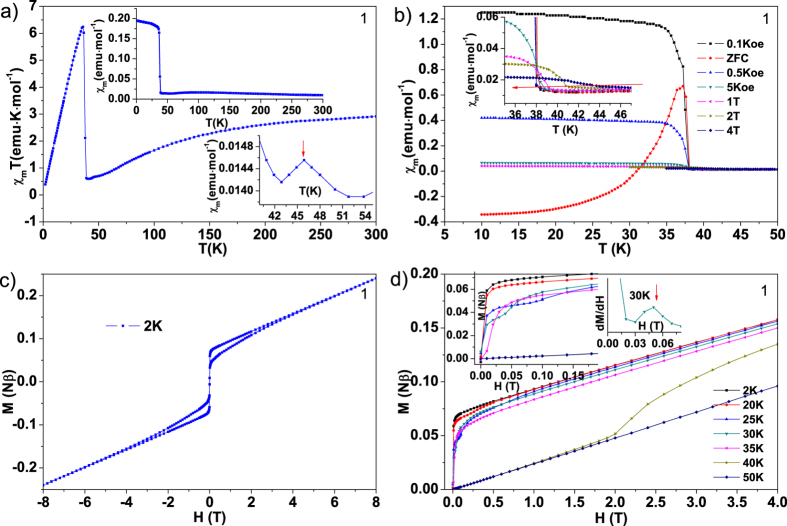
(**a**) Variable-temperature χ_m_T of **1**. Up inset: variable-temperature χ_m_ of **1**; Down inset: enlarged variable-temperature χ_m_ around the minor peak at 46 K. (**b**) Field dependence of the FC magnetization of **1** under 100 Oe, 500 Oe, 0.5 T, 1 T, 2 T and 4 T, and ZFC measured with 100 Oe. Inset: enlarged ZFC curves around the spin order transition. (**c**) Magnetic loop of **1** at 2 K. (**d**) Field (H) and temperature (T) dependence of the magnetization (M) of **1**. Left inset: enlarged MH curves at low field; Right inset: dM/dH curve at 30 K.

**Figure 5:**
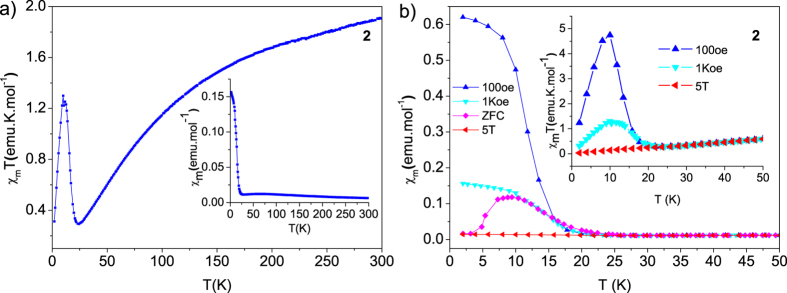
(**a**) variable-temperature χ_m_T of **2**, inset: variable-temperature χ_m_ of **2**. (**b**) Field dependence of the FC χ_m_ magnetization of **2** under 100 Oe, 1000 Oe and 5 T, and ZFC measured with 100 Oe, and relevant χ_m_T curves in the inset.

The variable-temperature magnetic susceptibilities for both complexes were measured in the range of 2–300 K under the external magnetic field of 1000 Oe (Fig. 4a for **1** and Fig. 5a for **2**). The χ_m_T value per Fe^2+^ and Co^2+^ units at room temperature is 2.91 and 1.91 emu·K·mol^−1^, respectively, which are very close to the spin only value of 3.0 emu·K·mol^−1^ for uncoupled high spin Fe^2+^ (*S* = 2) and 1.875 emu·K·mol^−1^ for uncoupled high spin Co^2+^ (*S* = 3/2) based on g = 2.00. For **1**, with decreasing temperature, the χ_m_T value of Fe^2+^ decreases gradually and attains the value of 0.60 emu·K·mol^−1^ at ~40 K, indicating the presence of antiferromagnetic coupling between the chloride-bridged Fe^2+^–Fe^2+^ with the transition temperature of ~92 K (Fig. S5). After that, the χ_m_T value starts to increase at a high speed and reaches its highest peak with the value about 6.24 emu·K·mol^−1^ at 36 K, and then decreases again to its lowest value 0.39 emu·K·mol^−1^ at 2.0 K.

The χ_m_T behavior of **2** (Fig. 5a) is similar to that of **1**, it also firstly decreases to the value of 0.29 emu·K·mol^−1^ at ~24 K, indicating the antiferromagnetic coupling between intrachain Co^2+^–Co^2+^ with the transition temperature of ~63 K (Fig. S5), then starts to increase and reaches its highest value of 1.30 emu·K·mol^−1^ at ~10 K, and then decreases again to its lowest value 0.31 emu·K·mol^−1^ at 2.0 K. The magnetic susceptibilities of Fe^2+^ and Co^2+^ conforms well to the Curie–Weiss law in the high temperature range of ~200–300 K and gives the negative Weiss constants −77 K for Fe^2+^ and −115 K for Co^2+^, and Curie constants 3.65 emu·K·mol^−1^ for Fe^2+^ and 2.61 emu·K·mol^−1^ for Co^2+^, demonstrating further the presence of antiferromagnetic alignments between intrachain spin centers in both complexes.

Upon further cooling after antiferromagnetic response at transition temperature of low dimensional correlation, the χ_m_T products of both complexes experience a remarkable rise and then rapidly decrease. The sharp rise of χ_m_T values of **1** at low temperature, with a very high maximum that is even far above the spin-only value expected for the Fe^2+^ unit, suggests the occurrence of long range spin order. This ferromagnetic-like magnetic response may still originate from noncollinear spin order in antiferromagnetically coupled systems, which can be attributed to incommensurate spiral spin order formed by partially frustrated trigonal spin lattice and will be explained in the section of spin Hamiltonian construction. It can be further supported by the field dependence of the field cooling (FC) magnetization since the noncollinear spin order is rather field-dependent. It can be seen from the field dependence of FC of **1** (Fig. 4b) that χ_m_ becomes weak at higher fields in the low temperature region, and the transition temperature changes gradually from ~44 K at 4 T to ~38 K at 100 Oe (inset of Fig. 4b). Zero Field cooling (ZFC) and FC curves of **1** measured under 100 Oe diverge at 37 K, confirming the onset of long-range order. The temperature dependence of χ_m_ of **1** (Right inset of Fig. 4a) also show a minor peak at ~46 K, implying a second spin order between the paramagnetic state and that incommensurate spiral spin order. For **2** (Fig. 5b), The FC χ_m_ curve approaches almost saturation below ~5 K and χ_m_T curves (inset of Fig. 5b) show a peak at ~10 K at 100 and 1000 Oe, suggesting an weak ferromagnetic-like order. This ferromagnetic-like order is further enhanced by the applied field of 5 T, where the maximum of χ_m_T almost disappears. As observed in Fig. 5b, the ZFC plot under a field of 100 Oe presents a cusp at around 10 K and shows disagreement with FC below that temperature, suggesting the onset of long-range order.

The noncollinear antiferromagnetic long-range order of **1** was further studied by the isothermal field dependence of the magnetizations at different temperatures below and above the critical temperature (~37 K) of transition (Fig. 4d). The step-wise M versus H curves at 2, 20, 25, 30 and 35 K exhibit field induced spin-flop-like metamagnetic transition. This step-wise magnetization becomes less pronounced upon decreasing the temperature, and the differentials of these curves show that the critical fields (200 Oe (35 K), 500 Oe (30 K), 1000 Oe (25 K) 2600 Oe (20 K) and 1.1 T (2 K)) shift to higher fields with decreasing temperature, indicating the phase transitions of metamagnetism. The magnetization of 0.25 Nβ at high field of 8 T and at 2 K (Fig. 4c) is much smaller than the saturation value of 4 Nβ for an isotropic high-spin Fe^2+^, in agreement with the antiferromagnetic coupling. Furthermore, the coercive field of close to 0 Oe at 2 K confirms the soft character of the long-rang order in **1**.

**Figure 6:**
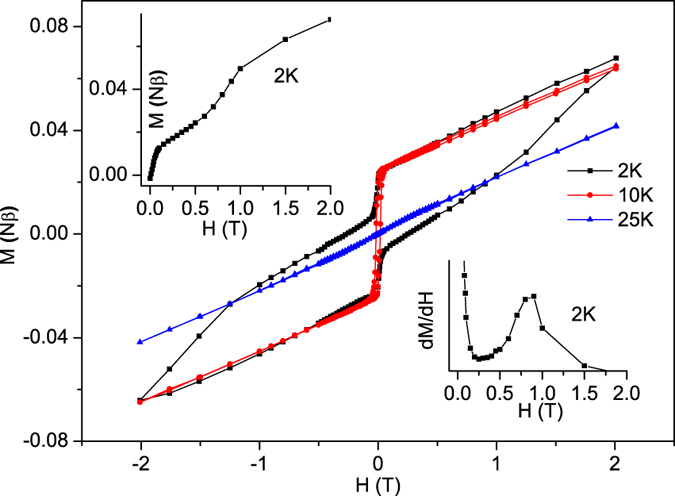
Magnetic loop of 2 at 2, 10 and 25 K. Left inset: field (H) dependence of magnetization (M) of **2**. Right inset: dM/dH curve at 2 K.

For **2**, the step-wise magnetization curve (Fig. 6, inset) at 2 K shows a critical field of 0.86 T, suggesting the presence of a metamagnetic transition. As is similar to **1**, The magnetization at 2 K and at the field of 2 T is 0.073 Nβ, which is far below the normally observed value of 3 Nβ for an isotropic high-spin Co^2+^, indicating the antiferromagnetic coupling.

**Figure 7:**
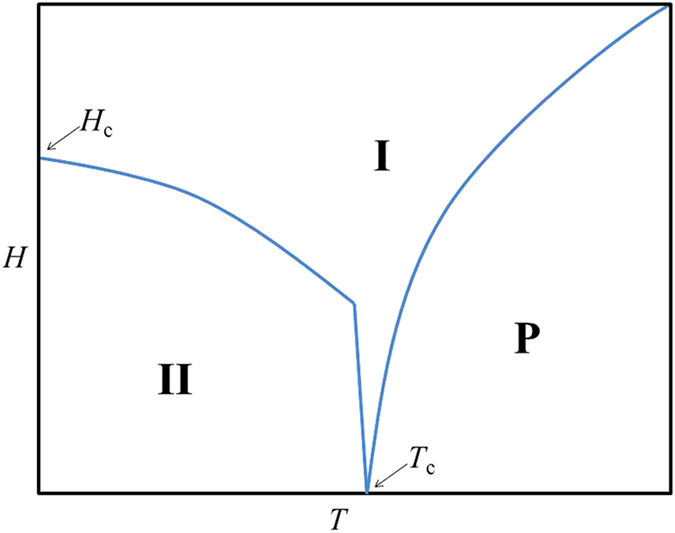
Schematic phase diagram of 1 and 2 based on the magnetic susceptibility measurement results. Paramagnetic phase (**P**) at high temperature, ground state phase (**II**) at low temperature and low magnetic field, and phase **I** between them. The critical temperature *T*_c_ is ~37 K for **1** and ~10 K for **2**, and the critical field *H*_c_ is ~1.1 T and ~0.86 T for **1** and **2**, respectively.

Based on the magnetic susceptibility measurement results, schematic magnetic *H*-*T* phase diagrams of both complexes can be built (Fig. 7). Obviously, paramagnetic phase (**P**) exist at high temperature and ground state phase **II** exist at low temperature. It can be seen from the experimental χ_m_-T curve of **1** (down inset of Fig. 4) that a very minor peak (~46 K) is located between the onset of ground state phase **II** (~37 K, Fig. S5) and transition temperature of low dimensional correlation (~92 K, Fig. S5), implying another phase **I** in the phase diagram, which however is not obviously detected by the magnetic susceptibility measurement for **2**. The field induced metamagnetic behavior found from experimental *M*-*H* curves of **1** and **2** at low temperatures should happen on the critical line between phases **II** and **I**, and the temperature drops down, the critical field should shift to large values, according to the temperature dependence of *M*-*H* curves of **1** (Inset of Fig. 4d); The field dependence of the FC magnetization of **1** (Fig. 4b) shows that the transition temperature changes gradually from ~44 K at 4 T to ~38 K at 100 Oe (inset of Fig. 4b), it can be attributed to the critical line between phases **P** and **I**.

### Spin Hamiltonian construction

In order to get insights into the magnetic properties of **1** and **2**, the band structures and density of states (DOS) of both complexes were calculated based on density-functional theory (DFT), and the magnetic exchange coupling constants in both structures were calculated by the energy-mapping method^27^.

**Figure 8:**
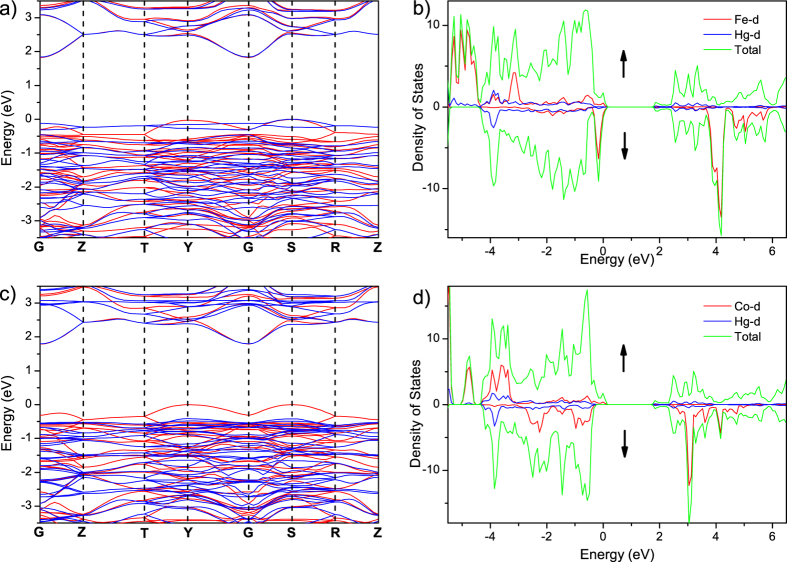
Band structures of up-spin (red line) and down-spin (blue line) for the ferromagnetic state of **1** (a) and **2** (c) along the high symmetry *k*-points: G(0, 0, 0), Z(0, 0, 0.5), T(0.5, 0.5, 0.5), Y(0.5, 0.5, 0), G(0, 0, 0), S(0, 0.5, 0), R(0, 0.5, 0.5), Z(0, 0, 0.5). Total and partial DOS for the Hg, Fe and Co atoms in **1** (**b**) and **2** (**d**). The Fermi level is set at 0 eV for all the band structures and DOS. The calculated band gaps of **1** and **2** are 1.83 and 1.79 eV, respectively.

The electronic structures of **1** and **2** calculated for the ferromagnetic state are presented in Fig. 8, which show that both ferromagnetic states are insulating with indirect band gaps. The calculated band gaps of **1** and **2** are 1.83 and 1.79 eV, which are close to the experimental values 1.93 and 1.64 eV, respectively. The calculated spin magnetic moments per Fe^2+^ and Co^2+^ are respective 3.74 and 2.70 *μ*_B_, which can be gotten from the difference between the integration of DOS of up and down spin, and they are consistent with the pictures of high-spin Fe^2+^ (*d*^6^, *S* = 2) and Co^2+^ (*d*^7^, *S* = 1.5) in **1** and **2**.

**Figure 9:**
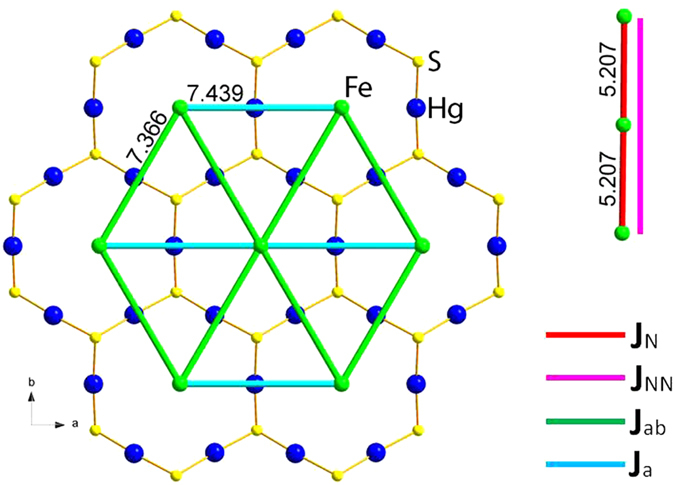
The relative locations of Fe^2+^ sites and Hg_3_S_2_ layer in **1** along the *c* direction, showing the interchain *J*_ab_ and *J*_a_ interactions (left) and intrachain *J*_N_ and *J*_NN_ interactions (right top). The Fe^2+^ plane is parallel to Hg_3_S_2_ layer with a shift of *c*/4 along the *c* direction. The assigned magnetic interactions in **2** is same to those in **1**.

From the experimental results we can see that the interchain interactions are crucial for the long-range spin order at low temperature and field induced metamagnetic transitions. Four magnetic exchange parameters are considered, including intrachain *J*_N_ and *J*_NN_, and interchain *J*_ab_ and *J*_a_ (Fig. 9), *J*_N_ is the strongest coupling between the nearest neighboring spin sites Fe^2+^ or Co^2+^ within the magnetic chains. The coupling *J*_NN_ between the next nearest neighboring spin sites within the chains were also included in the calculation to cover the possible minor but important interactions. The magnetic chains in both complexes are aligned trigonally along the *a* and *b* directions, Fe^2+^ and Co^2+^ sites are exactly in the *ab* plane. There are two independent interchain couplings in the *ab* plane due to a 2_1_ screw axis along the *c* direction, *J*_ab_ along the *a* ± *b* direction and *J*_a_ along the *a* direction.

**Figure 10:**
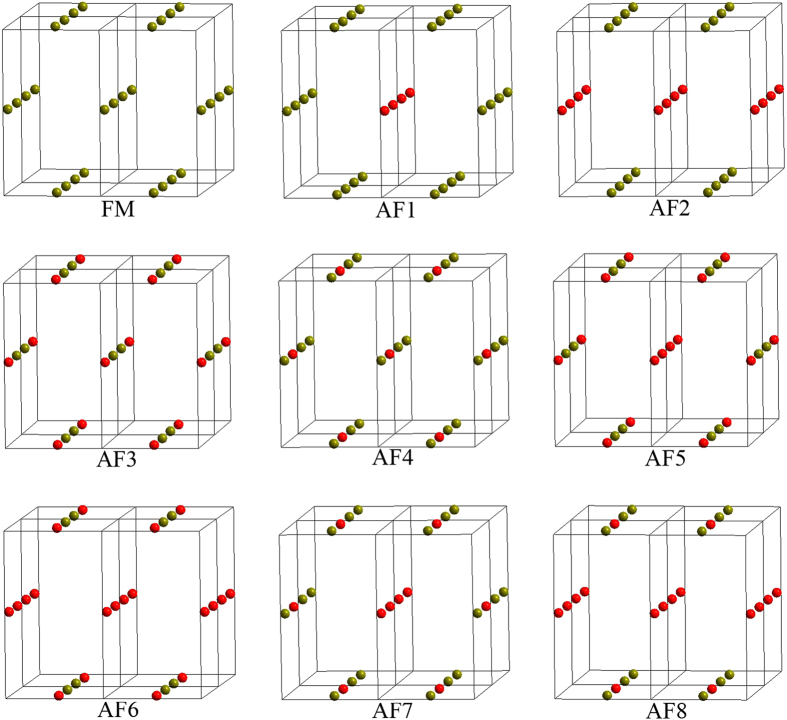
Nine ordered spin states employed to extract the four spin exchange parameters *J*_N_, *J*_NN_, *J*_ab_ and *J*_a_. For simplicity, only the magnetic cations Fe^2+^ or Co^2+^ are shown. The spin-up and spin-down magnetic sites are represented by dark yellow and red spheres, respectively.

The four magnetic exchange parameters *J*_N_, *J*_NN_, *J*_ab_ and *J*_a_ can be evaluated by examining the nine ordered spin states, among which four redundant spin states are added to check the consistency of calculation, i.e., the FM and AFn (n = 1–9) states, defined in Fig. 10 in terms of a 2 × 1 × 2 supercell. The total spin exchange interaction energies of the nine ordered spin states are expressed in terms of the Hamiltonian:


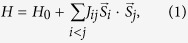


where *H*_0_ is related to non-spin variables, and is the same for all the nine spin states. *J*_ij_ = *J*_N_, *J*_NN_, *J*_ab_ and *J*_a_, is the spin exchange parameter for the spin exchange interaction between the spin sites *i* and *j*, while 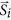 and 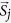 are the spin angular momentum at the spin sites *i* and *j*, respectively. The energy expressions of the nine ordered spin states obtained per 2 × 1 × 2 supercell (*S* = 2 for Fe^2+^ and *S* = 1.5 for Co^2+^) can be expressed as:


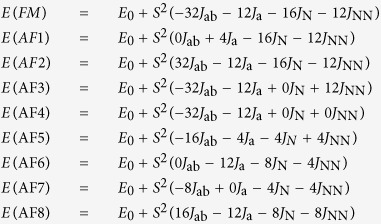


Table 2 summarizes the relative energies of the nine ordered magnetic states per 2 × 1 × 2 supercell, determined from our LDA + *U* calculations. When the relative energies of these spin states are mapped onto the corresponding energies determined from the spin Hamiltonian, the values of *J*_N_, *J*_NN_, *J*_ab_ and *J*_a_ can be obtained (Table 3).

It can be seen from the values of *J*_N_, *J*_NN_, *J*_ab_ and *J*_a_ based on LDA+*U* calculations that the intrachain spin exchanges *J*_N_ and interchain spin exchanges *J*_ab_ and *J*_a_, are antiferromagnetic, and *J*_ab_ and *J*_a_ are about one order weaker than *J*_N_. Although *J*_NN_ is ferromagnetic, it is about one order weaker than the interchain interactions. *J*_N_ is the strongest interaction among all the considered interactions, and its anisotropy may not be innegligible. The *J*_N_ along the *a, b* and *c* directions, i.e., *J*_Na_, *J*_Nb_ and *J*_Nc_, respectively, are extracted from LDA+*U*+SOC calculations of four broken-symmetry spin states for each of them based on the spin Hamiltonian 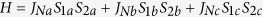
27, where *S*_1a_, *S*_1b_, *S*_1c_, *S*_2a_, *S*_2b_, and *S*_2c_ are spin angular momentum at the spin sites 1 and 2 in one primitive cell along the *a, b* and *c* directions, respectively. Primitive cell contains two magnetic sites and only one primitive cell is adopted in the LDA+*U*+SOC calculation. The spin directions of spin sites and absolute energy for every spin state are summarized in Table S3, and the calculated *J*_Na_, *J*_Nb_ and *J*_Nc_ for **1** and **2** are presented in Table 3. Compared with other interactions, the anisotropy of *J*_N_ is relatively very small, ~3 μeV for **1** and ~1 μeV for **2,** and can be negligible.

It is worth noting here that the spin sites in the *ab* plane is not fully frustrated due to a 2_1_ screw axis along the *c* direction, even if *J*_ab_ and *J*_a_ are very close to each other, complex **1** and **2** can therefore be regarded as an partially frustrated spin systems. Based on the calculated magnetic coupling parameters, it is reasonable to construct spin Hamiltonian of both systems only considering isotropic antimagnetic intrachain and interchain interactions as:


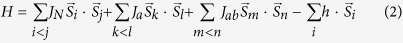


where the last sum describe the Zeeman energy of the spins in a magnetic field *h*. Magnetic dipole-dipole (MDD) interactions are not included in this Hamiltonian because the distance between the nearest spin sites in the magnetic chains (~5.3 Å) and in the *ab* plane (~7.3 Å) are relative large for both **1** and **2**, and the value of magnetic dipole-dipole interactions are estimated to be on the order of ~0.01 meV within the distance of ~11 Å based on 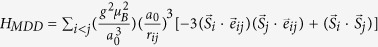, where *a*_0_ is the Bohr radius (0.529177 Å), *r*_ij_ is the distance between the spin sites *i* and *j*, and 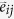 is the unit vector along the distance^28^.

The constructed spin Hamiltonian for spin systems **1** and **2** is the so-called row model that has been solved analytically using Landau-type approach by M. E. Zhitomirsky^29^,^30^. The ground state (phase **II** in Fig. 7) of Hamiltonian of both complexes at *h* = 0 is a spin helix, i.e., 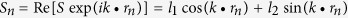, the ground state 120^o^ configuration of fully frustrated case (*J*_a_ = *J*_ab_) becomes unstable because of little difference between *J*_a_ (0.11 for **1** and 0.44 for **2**) and *J*_ab_ (0.12 for **1** and 0.46 for **2**). Minimizing the exchange energy with respect to *k* at magnetic field *h* = 0, the incommensurate ground state propagating along the direction 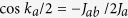 can be gotten. The spin configuration has locally triangular structure with the vector pair (***l***_1_, ***l***_2_) rotating by the small angle 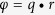, 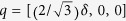, 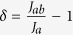 (0.091 or **1** and 0.045 for **2**). According to the analytical results, phase **I** in the phase diagram can be further divided into the incommensurate 120^o^ configuration and two linearly polarized commensurate phases.

In conclusion, two new inorganic supramolecular complexes, (Hg_3_S_2_)(FeCl_4_) (**1**) and (Hg_3_S_2_)(CoCl_4_) (**2**), in which 1-D infinite _∞_^1^(FeCl_4_)^2−^ or _∞_^1^(CoCl_4_)^2−^ chains penetrate perpendicularly the 2-D (Hg_3_S_2_)^2+^ layers through hexagonally aligned voids, assembled by Van der Waals forces, have been prepared by solid-state reactions. The comparison among those metal chalcohalides with similar low-dimensional structure features indicates semiconductive Hg_3_Q_2_ chalcogenide layers exhibit high flexibility to stabilize different kinds of guest polyanions. Both complexes feature long-range spin order below critical temperatures ~37 K for **1** and ~10 K for **2** with strong field dependence, indicated by variable-temperature magnetic susceptibilities. Field dependence of magnetic susceptibilities at low temperatures show that the both complexes exhibit field induced metamagnetic transitions. The intrachain and interchain magnetic coupling constants as well as the anisotropy of the nearest intrachain coupling of **1** and **2** were extracted by DFT+*U* and DFT+*U*+SOC calculations, showing that both nearest intrachain and interchain interactions are antiferromagnetic and dominated compared to next nearest ferromagnetic intrachain interactions, anisotropy of nearest intrachain interactions and magnetic dipole-dipole interactions. The both complexes can be regarded as partially frustrated trigonal spin systems caused by a small orthorhombic distortion which yields two nonequivalent exchange bonds in the *ab* plane. The spin Hamiltonian for the both complexes was constructed, and the ground state is proposed to be incommensurate spiral spin order, which has locally triangular structure with the vector pair (***l***_1_, ***l***_2_) rotating by the small angle 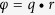, *q* = [0.105, 0, 0] for **1** and *q* = [0.052, 0, 0] for **2**.

The study indicates that cooperative magnetic ordering induced by partially geometric frustration can be realized in inorganic supramolecular systems assembled by weak van der Waals’ interactions, as is different from those frustrated systems formed by strong covalent^31^, and ionic bonding^32^. Considering the semiconductive character of metal chalcohalides especially those without toxic elements and the assembling flexibility with transition metal halides, some chalcohalides of transition metals may be predicted to exhibit interesting multifunctional semiconductive and cooperative magnetic properties.

## Methods

### Reagents and syntheses

All the starting materials were used as received without further purification. Complex **1** was crystallized from the reaction containing HgCl_2_ (1.0 mmol, 99.5%), Hg_2_Cl_2_ (1.0 mmol, 99.5%), iron powder (1.0 mmol, 99.99%), and sulfur powder (2.0 mmol, 99.999%). Complex **2** was crystallized from the reaction containing HgCl_2_ (1.0 mmol, 99.5%), Hg_2_Cl_2_ (1.0 mmol, 99.5%), cobalt powder (1.0 mmol, 99.9%), and sulfur powder (2.0 mmol, 99.999%). The starting materials were loaded into Pyrex tubes, evacuated to 1 × 10^−4^ Torr, and flame-sealed, then the tubes were heated in furnace from room temperature to 200 °C at a rate of 50 °C/h and kept at that temperature for 12 hours, then heated to 400 °C and 450 °C for **1** and **2**, respectively, at 20 °C/h, and kept at those temperatures for 3 days, then slowly cooled to 100 °C at a rate of 2.5 °C/h. Red crystals of **1** and blue crystals of **2** were obtained. The impurity of red amorphous solid solution can be found in the products of **1** and can be easily cleaned up mechanically.

### Crystal structure determination

The respective single crystals of **1** and **2** with dimensions of 0.20 × 0.20 × 0.10 mm^3^ and 0.20 × 0.10 × 0.05 mm^3^, respectively, were mounted on glass fiber for single-crystal X-ray diffraction analysis. The measurements were performed on a Rigaku Saturn 70 CCD diffractometer equipped with graphite-monochromated Mo K*α* radiation (*λ* = 0.71073 Å) at 293 K. The intensity data sets were collected with an *ω* scan technique and reduced using the CrystalClear software^33^. The structures of **1** and **2** were solved by direct methods and refined by full-matrix least-squares techniques on ***F***^2^. All of the calculations were performed with the Siemens SHELXL version 5 package of crystallographic software^34^.

### Powder XRD, Thermogravimertric Analysis (TGA), EDS and UV-Vis-NIR Diffuse Reflectance Spectroscopies

The powder XRD patterns (Fig. S1) were collected with a Rigaku DMAX 2500 diffractometer powered at 40 kV and 100 mA for Cu K*α* radiation (*λ* = 1.5406 Å) with a scan speed of 5°/min at room temperature. The simulated patterns were produced using the Mercury program and single-crystal reflection data. TGA studies of **1** and **2** were carried out with a NETZSCH STA 449C instruments under a nitrogen atmosphere. The samples and reference were held in Al_2_O_3_ crucibles, heated at a rate of 10 °C/min from room temperature to 600 °C. Semi quantitative microscope analysis using energy-dispersive X-ray spectroscopy (EDS) were performed on a JSM6700F scanning electron microscope (SEM). The diffuse reflectance spectra were recorded at room temperature on a computer-controlled Lambda 900 UV-Vis-NIR spectrometer equipped with an integrating sphere in the wavelength range of 300–2000 nm. A BaSO_4_ plate was used as a reference, on which the finely ground powders of the samples were coated. The absorption spectra were calculated from reflection spectra using the Kubelka-Munk function^35^.

### Magnetic Measurements

Magnetic susceptibilities of randomly oriented polycrystalline samples of **1** and **2** were measured in the temperature range of 2–300 K on a Quantum Design MPMS(SQUID)-XL magnetometer. The magnetic responses were corrected with diamagnetic blank data of the sample holder measured separately, and the diamagnetic contributions to the both complexes were estimated from Pascal’s constants.

### First-principles calculations

Spin-polarized DFT calculations employed the projector augmented wave method encoded in the Vienna *ab initio* simulation package^36^,^37^, the local density approximation (LDA), and the plane wave cutoff energy of 500 eV. The LDA plus on-site repulsion *U* method LD + *U*^38^, was employed to properly describe the electron correlation associated with the Fe and Co 3*d* states. The suitable *U* was chosen by comparing the calculated band gaps using different *U* from 3.0 to 7.0 eV with experimental ones. It can be seen from Table 4 that band gaps using *U* = 5.0, 6.0 and 7.0 eV agree well with the experimental value of **1**, and band gaps using *U* = 3.0, 4.0 and 5.0 agree well with the experimental value of **2**. The *U* of 5.0 eV^39^,^40^, was chosen for the further calculation.

Anisotropic exchange interaction constants of nearest (strongest) neighboring intrachain Fe−Fe and Co−Co interactions were extracted from energies of different spin configurations considering the spin-orbit coupling (SOC) effect, while the interchain interactions were treated to be isotropic. It is worth noting here that the values of *U* don’t affect spin exchange parameters and the spin Hamiltonian. Taking *U* of 5.0 and 7.0 eV as example, as can be seen from Table 5, Although all exchange parameters using *U* = 5.0 eV are much larger than respective values using *U* = 7.0 eV, the difference between *J*_N_, *J*_NN_, *J*_ab_ and *J*_a_ of both complexes were found to be almost same, i.e., antiferromagnetic *J*_ab_ and *J*_a_ are about one order weaker than *J*_N_, and ferromagnetic *J*_NN_ is about one order weaker than *J*_ab_ and *J*_a_. Similar ground spin state and phase diagram can be derived from the calculated exchange parameters using different *U*.

## Additional Information

**How to cite this article**: Jiang, X.-M. *et al.* Partial Geometric Frustration in Inorganic Supramolecular Spin Systems with One-Dimensional Trigonally Aligned Magnetic Chains _∞_^1^(MCl_4_)^2−^ (M = Fe^2+^, Co^2+^). *Sci. Rep.* **5**, 17344; doi: 10.1038/srep17344 (2015).

## Supplementary Material

Supplementary Information

## Figures and Tables

**Table 1:** Crystal data and structure refinement parameters of **1** and **2**.

Chemical formula	(Hg_3_S_2_)(FeCl_4_) (1)	(Hg_3_S_12_)(CoCl_4_) (2)
Fw	863.54	866.62
Crystal system	Orthorhombic	Orthorhombic
Space group	*C*222(1)	*C*222(1)
*a* (Å)	7.4388(9)	7.342(4)
*b* (Å)	12.7160(16)	12.734(6)
*c* (Å)	10.4145(14)	10.635(5)
*V* (Å^3^)	985.1(2)	994.3(8)
*Z*	4	4
*D*_calcd_ (g cm^−1^)	5.822	5.789
*μ* (mm^−1^)	49.482	49.235
*F*(000)	1464	1468
*θ* range (˚)	3.17–25.46	3.20 –25.48
Index range	−8≤h≤8	−8≤h≤8
	−13≤k≤15	−15≤k≤14
	−12≤l≤12	−12≤l≤12
Measd. Reflns	3552	2255
Indep. reflns/*R*_int_	913/0.1225	868/0.1002
Obsd reflns	655	431
*R*1^a^ (*I* > 2*σ*(*I*))	0.0570	0.0742
*wR*2^b^ (all data)	0.1342	0.1395
GOF on *F*^2^	0.905	0.940
Δ*ρ*_max_/Δ*ρ*_min_, e/Å^3^	2.265/−1.668	2.699/−2.534

^a^*R*1 = ||*F*_o_| – |*F*_c_||/|F_o_|

^b^*wR*^2^ = [*w*(*F*_o_^2^ – *F*_c_^2^)^2^]/[*w*(*F*_o_^2^)^2^]^1/2^.

**Table 2:** Relative energies in meV of the nine ordered spin states of **1** and **2** determined from LDA+*U* calculations.

	*E*(FM)	*E*(AF1)	*E*(AF2)	*E*(AF3)	*E*(AF4)	*E*(AF5)	*E*(AF6)	*E*(AF7)	*E*(AF8)
**1**	0	−23.27	−32.09	−180.73	−182.20	−146.80	−105.86	−153.69	−114.59
**2**	0	−49.56	−67.56	−219.04	−220.73	−188.38	−142.35	−202.02	−160.04

**Table 3:** Values of the spin exchange parameters for **1** and **2** in meV extracted from the LDA+*U* and LDA+*U*+SOC calculations.

		LDA+*U*			LDA+*U*+SOC		
	*J*_ab_	*J*_a_	*J*_N_	*J*_NN_	*J*_Na_	*J*_Nb_	*J*_Nc_
**1**	0.124	0.114	2.860	−0.026	2.859	2.862	2.859
**2**	0.464	0.440	6.151	−0.051	6.150	6.151	6.151

**Table 4:** The calculated band gaps (eV) of **1** and **2** using different *U*.

*U*	3.0	4.0	5.0	6.0	7.0	Experimental value
Complex **1**	1.13	1.51	1.83	1.96	2.08	1.93
Complex **2**	1.51	1.67	1.79	1.91	2.00	1.64

**Table 5:** The calculated four spin exchange parameters *J*_N_, *J*_NN_, *J*_ab_ and *J*_a_ of 1 and 2 in meV using *U *= 5.0 and 7.0 eV.

Complex 1	*J*_ab_	*J*_a_	*J*_N_	*J*_NN_
*U* = 5.0	0.124	0.114	2.860	−0.026
*U* = 7.0	0.029	0.027	0.921	−0.005
Complex **2**				
*U* = 5.0	0.464	0.440	6.151	−0.051
*U* = 7.0	0.130	0.124	2.197	−0.006
