# Supplementary material for: Partial Geometric Frustration in Inorganic Supramolecular Spin Systems with One-Dimensional Trigonally Aligned Magnetic Chains ∞1(MCl4)2− (M = Fe2+, Co2+)
Source: Sci Rep. 2015 Dec 9;5:17344. doi: 10.1038/srep17344 (PMC4673534; doi:10.1038/srep17344)
Supplement: Supplementary Information [file srep17344-s1.pdf]

Supporting Information

Partial Geometric Frustration in Inorganic Supramolecular Spin Systems with One-Dimensional Trigonally Aligned Magnetic Chains $_\infty{}^1(MCl_4)^{2-}$ (M = Fe^{2+}, Co^{2+})

Xiao-Ming Jiang, Xiao-Guo Li, Ming-Jian Zhang, Zhi-Fa Liu, Yong Liu, Jun-Ming Liu, and Guo-Cong Guo

Table S1. Atomic coordinates and equivalent isotropic displacement parameters ($Å^2$) for **1** and **2**.

Atom	x	y	z	$U_{(eq)}{}^a$ ($Å^2$)	Occupancy	Wyckoff
			**1**			
Hg(1)	0.46335(10)	0.5000	0.5000	0.0440(3)	1	4a
Hg(2)	0.21496(7)	0.25171(5)	0.48853(7)	0.03950(17)	1	8c
Fe(1)	0.0000	0.4992(3)	0.2500	0.0223(7)	1	4b
S(1)	0.9755(5)	0.1675(3)	0.5956(4)	0.0271(10)	1	8c
Cl(1)	0.5483(7)	0.0000	0.5000	0.0495(18)	1	4a
Cl(2)	0.0000	0.3206(5)	0.2500	0.0429(18)	1	4b
Cl(3)	0.2307(5)	0.0854(3)	0.2684(4)	0.0325(11)	1	8c
			**2**			
Hg(1)	0.48313(9)	0.5000	0.5000	0.0648(3)	1	4a
Hg(2)	0.23501(6)	0.24972(6)	0.49258(8)	0.05608(18)	1	8c
Co(1)	0.0000	0.5010(3)	0.7500	0.0506(9)	1	4b
S(1)	0.9914(4)	0.1671(3)	0.5958(3)	0.0344(9)	1	8c
Cl(1)	0.5314(7)	0.0000	0.5000	0.104(3)	1	4a
Cl(2)	0.0000	0.3248(4)	0.2500	0.0430(15)	1	4b
Cl(3)	0.2356(3)	0.0880(2)	0.2600(4)	0.0329(9)	1	8c

${}^aU_{(eq)}$ is defined as one-third of the trace of the orthogonalized U_{ij} tensor.

Table S2. Select bond distances lengths (Å)^a for **1** and **2**.

bond	distance	bond	distance
	**1**		
Hg(1)-S(1)#1	2.353(4)	Fe(1)-Cl(1)#5	2.6284(8)
Hg(1)-S(1)#2	2.353(4)	S(1)-Hg(1)#6	2.353(4)
Hg(2)-S(1)#3	2.359(4)	S(1)-Hg(2)#7	2.359(4)

Hg(2)-S(1)#2	2.362(4)	S(1)-Hg(2)#8	2.362(4)
Fe(1)-Cl(2)	2.271(7)	Cl(1)-Fe(1)#6	2.6284(8)
Fe(1)-Cl(3)#4	2.292(4)	Cl(1)-Fe(1)#9	2.6284(8)
Fe(1)-Cl(3)#1	2.292(4)	Cl(3)-Fe(1)#6	2.292(4)
Fe(1)-Cl(1)#1	2.6284(8)		
	**2**		
Hg(1)-S(1)#1	2.360(4)	Co(1)-Cl(1)#2	2.6687(13)
Hg(1)-S(1)#2	2.360(4)	S(1)-Hg(2)#6	2.348(3)
Hg(2)-S(1)#3	2.348(3)	S(1)-Hg(2)#7	2.355(3)
Hg(2)-S(1)#1	2.355(3)	S(1)-Hg(1)#8	2.360(4)
Co(1)-Cl(2)#4	2.218(6)	Cl(1)-Co(1)#9	2.6687(13)
Co(1)-Cl(3)#1	2.251(3)	Cl(1)-Co(1)#8	2.6687(13)
Co(1)-Cl(3)#5	2.251(3)	Cl(2)-Co(1)#10	2.218(6)
Co(1)-Cl(1)#5	2.6687(13)	Cl(3)-Co(1)#9	2.251(3)

[a] Symmetry codes. Compound **1**: (#1) x-1/2,y+1/2,z; (#2) x-1/2,-y+1/2,-z+1; (#3) x-1,y,z; (#4) -x+1/2,y+1/2,-z+1/2; (#5) -x+1/2,-y+1/2,z-1/2; (#6) x+1/2,y-1/2,z. Compound **2**: (#1) x-1/2,-y+1/2,-z+1; (#2) x-1/2,y+1/2,z; (#3) x-1,y,z; (#4) -x,-y+1,z+1/2; (#5) -x+1/2,-y+1/2,z+1/2; (#6) x+1,y,z; (#7) x+1/2,-y+1/2,-z+1; (#8) x+1/2,y-1/2,z ; (#9) -x+1/2,-y+1/2,z-1/2; (#10) -x,-y+1,z-1/2.

Table S3. The spin directions of two spin sites (Site 1 and Site 2) in one primitive cell for four ordered spin states (State 1 to 4) in the DFT +U + SOC calculation of J_{Na}, J_{Nb} and J_{Nc} in complexes **1** and **2**, and the calculated absolute energy (eV) for each spin states.

	State 1	State 2	State 3	State 4
	J_{Na}*			
Site 1	(1 0 0)	(1 0 0)	(-1 0 0)	(-1 0 0)
Site 2	(1 0 0)	(-1 0 0)	(1 0 0)	(-1 0 0)
Energy(**1**)	E1=57.559359	E2=57.604706	E3=57.604706	E4=57.559225
Energy(**2**)	E1=53.792008	E2=53.846699	E3=53.846699	E4=53.792020
	J_{Nb}			
Site 1	(0 1 0)	(0 1 0)	(0 -1 0)	(0 -1 0)
Site 2	(0 1 0)	(0 -1 0)	(0 1 0)	(0 -1 0)
Energy(**1**)	E1=57.557849	E2=57.603247	E3=57.603248	E4=57.557750
Energy(**2**)	E1=53.792020	E2=53.846719	E3=53.846719	E4=53.792025
	J_{Nc}			
Site 1	(0 0 1)	(0 0 1)	(0 0 -1)	(0 0 -1)
Site 2	(0 0 1)	(0 0 -1)	(0 0 1)	(0 0 -1)
Energy(**1**)	E1=57.558393	E2=57.603742	E3=57.603743	E4=57.558276
Energy(**2**)	E1=53.789227	E2=53.843927	E3=53.843927	E4=53.789232

* J_{Na}, J_{Nb} and J_{Nc} are calculated by (E1+E4-E2-E3)/8 for **1** (S=2) and (E1+E4-E2-E3)/6 for **2** (S=3/2).

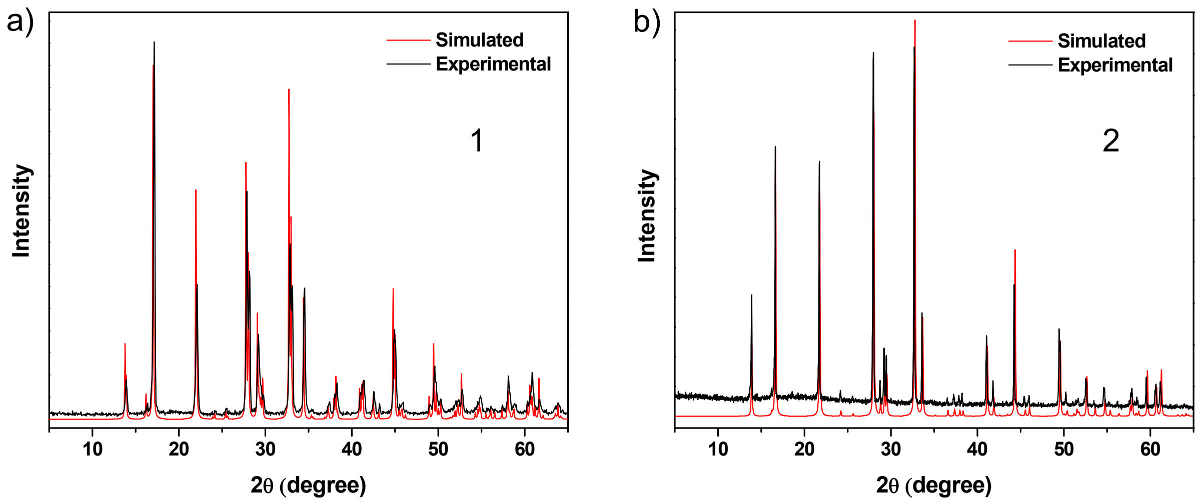

Figure S1. Simulated and experimental (~300 K) XRD powder patterns of **1** (a) and **2** (b).

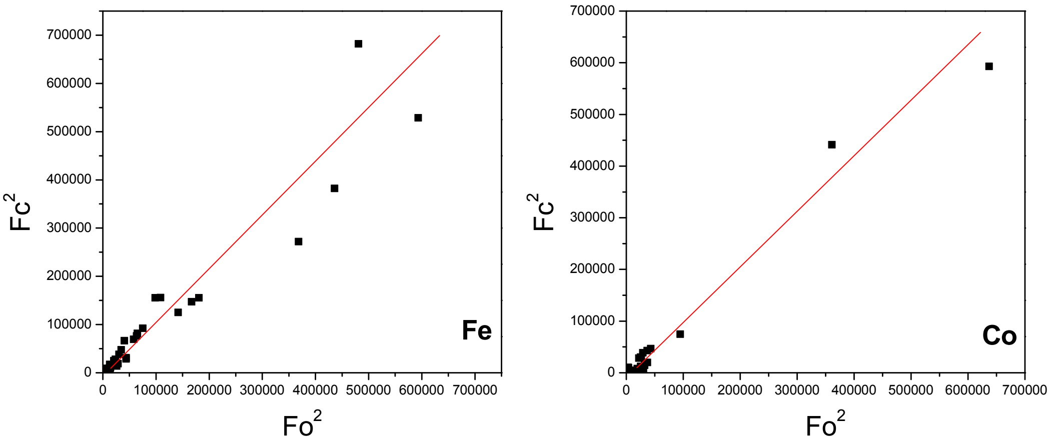

Figure S2. F_{obs}^2 vs F_{calc}^2 plots of the most 50 disagreeable reflections for **1** (left) and **2** (right).

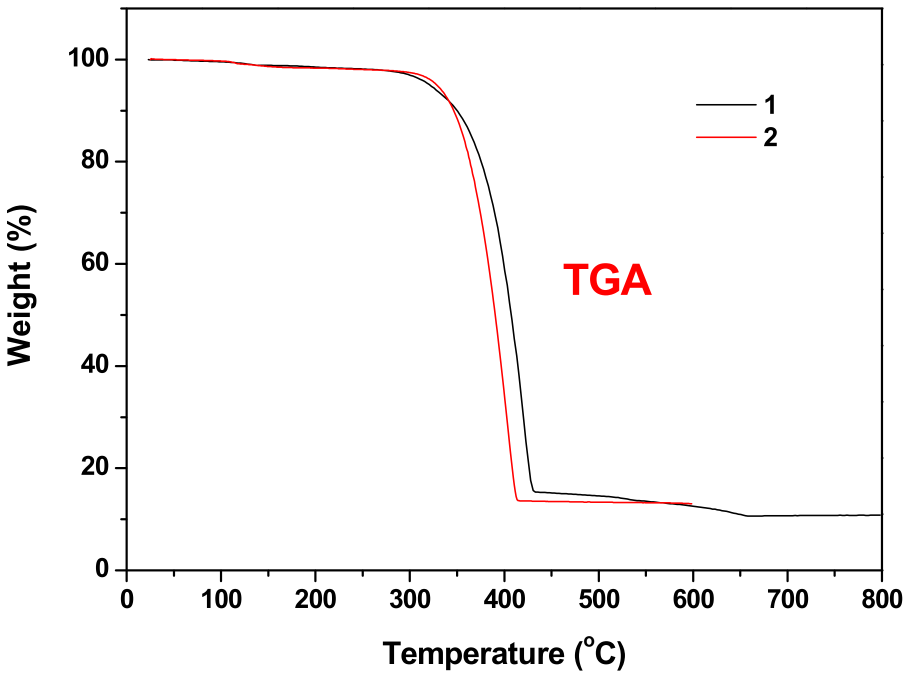

Figure S3. TGA spectra of **1** and **2.**

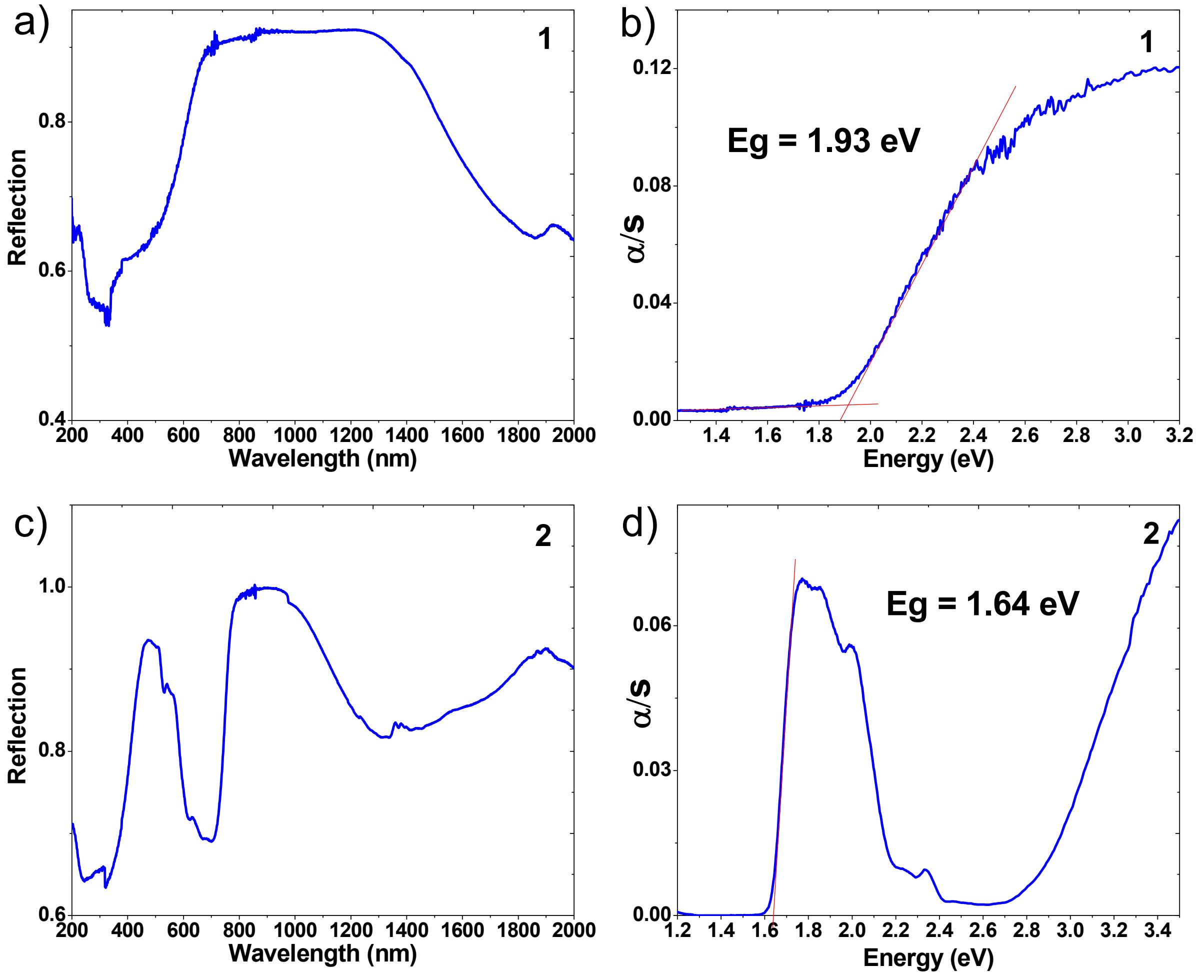

Figure S4. UV diffuse reflectance spectra of **1** (a) and **2** (c), and absorption spectra of **1** (b) and **2** (d).

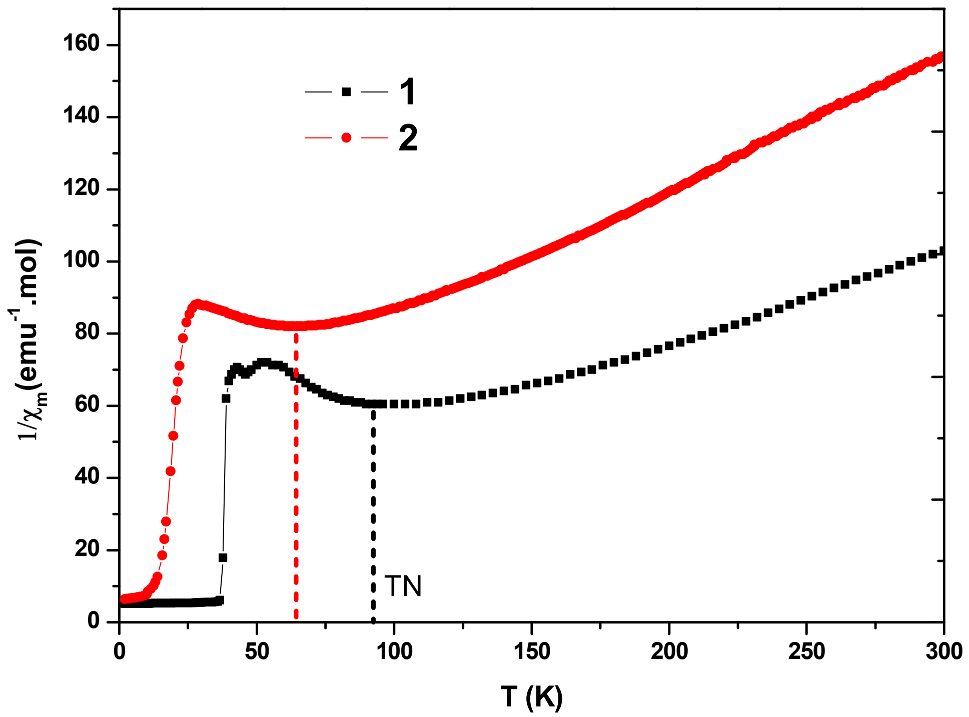

Figure S5. Temperature dependence of $1/\chi_m$ measured at the field of 1000 Oe, the transition temperature of low dimensional correlation of **1** and **2** are ~92 and ~63 K, respectively.

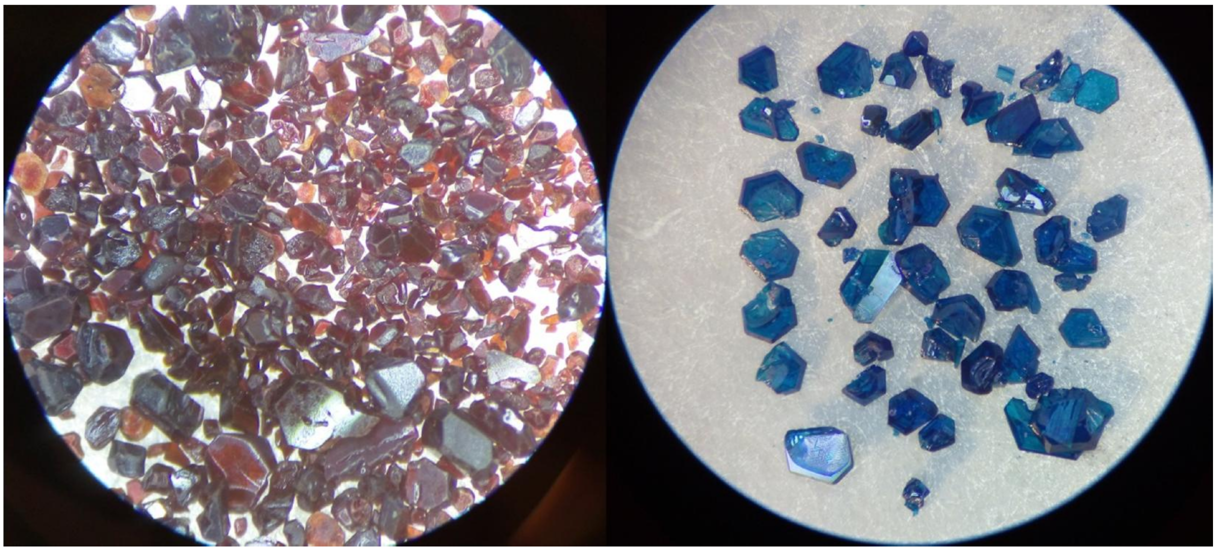

Figure S6. Photographs of single crystals of **1** (left) and **2** (right).

Below is the cifs of complexes **1** and **2**.

```
data_Cl4FeHg3S2

_audit_creation_method           SHELXL-97
_chemical_name_systematic
;
 'Cl4 Fe Hg3 S2'
;
_chemical_name_common             'Cl4 Fe Hg3 S2'
_chemical_melting_point          'not measured'
_chemical_formula_moiety          'Cl4 Fe Hg3 S2'
_chemical_formula_sum
 'Cl4 Fe Hg3 S2'
_chemical_formula_weight          863.54

loop_
 _atom_type_symbol
 _atom_type_description
 _atom_type_scat_dispersion_real
 _atom_type_scat_dispersion_imag
 _atom_type_scat_source
 'S'  'S'   0.1246   0.1234
 'International Tables Vol C Tables 4.2.6.8 and 6.1.1.4'
 'Cl'  'Cl'   0.1484   0.1585
 'International Tables Vol C Tables 4.2.6.8 and 6.1.1.4'
 'Fe'  'Fe'   0.3463   0.8444
 'International Tables Vol C Tables 4.2.6.8 and 6.1.1.4'
 'Hg'  'Hg'  -2.3894   9.2266
 'International Tables Vol C Tables 4.2.6.8 and 6.1.1.4'

_symmetry_cell_setting           'Orthorhombic'
_symmetry_space_group_name_H-M    'C222(1) '
_symmetry_Int_Tables_number       20

loop_
 _symmetry_equiv_pos_as_xyz
 'x, y, z'
 '-x, -y, z+1/2'
 '-x, y, -z+1/2'
 'x, -y, -z'
 'x+1/2, y+1/2, z'
 '-x+1/2, -y+1/2, z+1/2'
 '-x+1/2, y+1/2, -z+1/2'
 'x+1/2, -y+1/2, -z'

_cell_length_a                   7.4388(9)
_cell_length_b                   12.7160(16)
_cell_length_c                   10.4145(14)
_cell_angle_alpha                  90.00
_cell_angle_beta                  90.00
_cell_angle_gamma                   90.00
_cell_volume                     985.1(2)
_cell_formula_units_Z            4
_cell_measurement_temperature     293(2)
_cell_measurement_reflns_used     ?
_cell_measurement_theta_min       ?
_cell_measurement_theta_max       ?

_exptl_crystal_description        'plate'
_exptl_crystal_colour            'Red'
_exptl_crystal_size_max           0.2
_exptl_crystal_size_mid           0.2
_exptl_crystal_size_min           0.1
_exptl_crystal_density_meas       'not measured'
_exptl_crystal_density_diffrn     5.822
```

_exptl_crystal_density_method 'not measured'
_exptl_crystal_F_000 1464
_exptl_absorpt_coefficient_mu 49.482
_exptl_absorpt_correction_type 'Multi-scan'
_exptl_absorpt_correction_T_min 0.5375
_exptl_absorpt_correction_T_max 1.0000
_exptl_absorpt_process_details ?

_exptl_special_details
;
?
;

_diffrn_ambient_temperature 293(2)
_diffrn_radiation_wavelength 0.71073
_diffrn_radiation_type MoK\a
_diffrn_radiation_source 'fine-focus sealed tube'
_diffrn_radiation_monochromator graphite
_diffrn_measurement_device_type 'Rigaku SATURN70'
_diffrn_measurement_method 'CCD_Profile_fitting'
_diffrn_detector_area_resol_mean 13.6612
_diffrn_standards_number ?
_diffrn_standards_interval_count ?
_diffrn_standards_interval_time ?
_diffrn_standards_decay_% ?
_diffrn_reflns_number 3552
_diffrn_reflns_av_R_equivalents 0.1225
_diffrn_reflns_av_sigmaI/netI 0.1204
_diffrn_reflns_limit_h_min -8
_diffrn_reflns_limit_h_max 8
_diffrn_reflns_limit_k_min -13
_diffrn_reflns_limit_k_max 15
_diffrn_reflns_limit_l_min -12
_diffrn_reflns_limit_l_max 12
_diffrn_reflns_theta_min 3.17
_diffrn_reflns_theta_max 25.46
_reflns_number_total 913
_reflns_number_gt 655
_reflns_threshold_expression >2sigma(I)

_computing_data_collection 'CrystalClear (Rigaku Corp.,2007)'
_computing_cell_refinement 'CrystalClear (Rigaku Corp.,2007)'
_computing_data_reduction 'CrystalClear (Rigaku Corp.,2007)'
_computing_structure_solution 'SHELXS-97 (Sheldrick, 1990)'
_computing_structure_refinement 'SHELXL-97 (Sheldrick, 1997)'
_computing_molecular_graphics 'DIAMOND (Brandenburg, 1999)'
_computing_publication_material 'Siemens SHELXTL^TM^ 5'

_refine_special_details
;
Refinement of F^2^ against ALL reflections. The weighted R-factor wR and
goodness of fit S are based on F^2^, conventional R-factors R are based
on F, with F set to zero for negative F^2^. The threshold expression of
F^2^ > 2sigma(F^2^) is used only for calculating R-factors(gt) etc. and is
not relevant to the choice of reflections for refinement. R-factors based
on F^2^ are statistically about twice as large as those based on F, and R-
factors based on ALL data will be even larger.
;

_refine_ls_structure_factor_coef Fsqd
_refine_ls_matrix_type full
_refine_ls_weighting_scheme calc
_refine_ls_weighting_details
'calc w=1/[\s^2^(Fo^2^)+(0.0572P)^2^+0.0000P] where P=(Fo^2^+2Fc^2^)/3'
_atom_sites_solution_primary direct
_atom_sites_solution_secondary difmap
_atom_sites_solution_hydrogens ?

```
_refine_ls_hydrogen_treatment     ?
_refine_ls_extinction_method      SHELXL
_refine_ls_extinction_coef        0.00166(17)
_refine_ls_extinction_expression
 'Fc^*^=kFc[1+0.001xFc^2^\l^3^/sin(2\q)]^-1/4^'
_refine_ls_abs_structure_details
 'Flack H D (1983), Acta Cryst. A39, 876-881'
_refine_ls_abs_structure_Flack    0.50(4)
_refine_ls_number_reflns          913
_refine_ls_number_parameters      49
_refine_ls_number_restraints      0
_refine_ls_R_factor_all           0.0661
_refine_ls_R_factor_gt            0.0570
_refine_ls_wR_factor_ref          0.1342
_refine_ls_wR_factor_gt           0.1308
_refine_ls_goodness_of_fit_ref    0.905
_refine_ls_restrained_S_all       0.905
_refine_ls_shift/su_max           0.000
_refine_ls_shift/su_mean          0.000

loop_
 _atom_site_label
 _atom_site_type_symbol
 _atom_site_fract_x
 _atom_site_fract_y
 _atom_site_fract_z
 _atom_site_U_iso_or_equiv
 _atom_site_adp_type
 _atom_site_occupancy
 _atom_site_site_symmetry_order
 _atom_site_calc_flag
 _atom_site_refinement_flags_posn
 _atom_site_refinement_flags_adp
 _atom_site_refinement_flags_occupancy
 _atom_site_disorder_assembly
 _atom_site_disorder_group
Hg1 Hg 0.46335(10) 0.5000 0.5000 0.0440(3) Uani 1 2 d S T P . .
Hg2 Hg 0.21496(7) 0.25171(5) 0.48853(7) 0.03950(17) Uani 1 1 d . . . . .
Fe1 Fe 0.0000 0.4992(3) 0.2500 0.0223(7) Uani 1 2 d S T P . .
S1 S 0.9755(5) 0.1675(3) 0.5956(4) 0.0271(10) Uani 1 1 d . . . . .
Cl1 Cl 0.5483(7) 0.0000 0.5000 0.0495(18) Uani 1 2 d S T P . .
Cl2 Cl 0.0000 0.3206(5) 0.2500 0.0429(18) Uani 1 2 d S T P . .
Cl3 Cl 0.2307(5) 0.0854(3) 0.2684(4) 0.0325(11) Uani 1 1 d . . . . .

loop_
 _atom_site_aniso_label
 _atom_site_aniso_U_11
 _atom_site_aniso_U_22
 _atom_site_aniso_U_33
 _atom_site_aniso_U_23
 _atom_site_aniso_U_13
 _atom_site_aniso_U_12
Hg1 0.0370(3) 0.0233(4) 0.0717(6) -0.0002(7) 0.000 0.000
Hg2 0.0217(2) 0.0393(3) 0.0575(4) 0.0000(5) -0.0002(3) -0.0083(2)
Fe1 0.0119(10) 0.0166(13) 0.0383(17) 0.000 -0.0006(9) 0.000
S1 0.0289(17) 0.038(2) 0.0140(17) 0.001(2) -0.0022(14) -0.0002(18)
Cl1 0.042(2) 0.090(4) 0.016(3) -0.004(4) 0.000 0.000
Cl2 0.058(3) 0.015(3) 0.056(4) 0.000 -0.029(2) 0.000
Cl3 0.0220(17) 0.030(2) 0.046(2) -0.0091(17) -0.0053(15) 0.0103(16)

_geom_special_details
;
 All esds (except the esd in the dihedral angle between two l.s. planes)
 are estimated using the full covariance matrix.  The cell esds are taken
 into account individually in the estimation of esds in distances, angles
 and torsion angles; correlations between esds in cell parameters are only
 used when they are defined by crystal symmetry.  An approximate (isotropic)
```

treatment of cell esds is used for estimating esds involving l.s. planes.
;

loop_
 _geom_bond_atom_site_label_1
 _geom_bond_atom_site_label_2
 _geom_bond_distance
 _geom_bond_site_symmetry_2
 _geom_bond_publ_flag
Hg1 S1 2.353(4) 5_455 ?
Hg1 S1 2.353(4) 8_456 ?
Hg2 S1 2.359(4) 1_455 ?
Hg2 S1 2.362(4) 8_456 ?
Fe1 Cl2 2.271(7) . ?
Fe1 Cl3 2.292(4) 7 ?
Fe1 Cl3 2.292(4) 5_455 ?
Fe1 Cl1 2.6284(8) 5_455 ?
Fe1 Cl1 2.6284(8) 6_554 ?
S1 Hg1 2.353(4) 5_545 ?
S1 Hg2 2.359(4) 1_655 ?
S1 Hg2 2.362(4) 8_556 ?
Cl1 Fe1 2.6284(8) 5_545 ?
Cl1 Fe1 2.6284(8) 6 ?
Cl3 Fe1 2.292(4) 5_545 ?

loop_
 _geom_angle_atom_site_label_1
 _geom_angle_atom_site_label_2
 _geom_angle_atom_site_label_3
 _geom_angle
 _geom_angle_site_symmetry_1
 _geom_angle_site_symmetry_3
 _geom_angle_publ_flag
S1 Hg1 S1 175.62(17) 5_455 8_456 ?
S1 Hg2 S1 172.99(10) 1_455 8_456 ?
Cl2 Fe1 Cl3 118.59(14) . 7 ?
Cl2 Fe1 Cl3 118.59(14) . 5_455 ?
Cl3 Fe1 Cl3 122.8(3) 7 5_455 ?
Cl2 Fe1 Cl1 90.23(10) . 5_455 ?
Cl3 Fe1 Cl1 87.78(15) 7 5_455 ?
Cl3 Fe1 Cl1 92.00(15) 5_455 5_455 ?
Cl2 Fe1 Cl1 90.23(10) . 6_554 ?
Cl3 Fe1 Cl1 92.00(15) 7 6_554 ?
Cl3 Fe1 Cl1 87.78(15) 5_455 6_554 ?
Cl1 Fe1 Cl1 179.54(19) 5_455 6_554 ?
Hg1 S1 Hg2 103.86(15) 5_545 1_655 ?
Hg1 S1 Hg2 101.84(14) 5_545 8_556 ?
Hg2 S1 Hg2 104.29(16) 1_655 8_556 ?
Fe1 Cl1 Fe1 164.3(2) 5_545 6 ?

_diffrn_measured_fraction_theta_max   0.994
_diffrn_reflns_theta_full   25.46
_diffrn_measured_fraction_theta_full   0.994
_refine_diff_density_max   2.265
_refine_diff_density_min   -1.668
_refine_diff_density_rms   0.564

data_Cl4CoHg3S2

_audit_creation_method   SHELXL-97
_chemical_name_systematic
;
 'Cl4 Co Hg3 S2'
;

```
_chemical_name_common            'Cl4 Co Hg3 S2'
_chemical_melting_point           'not measured'
_chemical_formula_moiety         'Cl4 Co Hg3 S2'
_chemical_formula_sum
 'Cl4 Co Hg3 S2'
_chemical_formula_weight          866.62

loop_
 _atom_type_symbol
 _atom_type_description
 _atom_type_scat_dispersion_real
 _atom_type_scat_dispersion_imag
 _atom_type_scat_source
 'S'  'S'   0.1246   0.1234
 'International Tables Vol C Tables 4.2.6.8 and 6.1.1.4'
 'Cl'  'Cl'   0.1484   0.1585
 'International Tables Vol C Tables 4.2.6.8 and 6.1.1.4'
 'Co'  'Co'   0.3494   0.9721
 'International Tables Vol C Tables 4.2.6.8 and 6.1.1.4'
 'Hg'  'Hg'  -2.3894   9.2266
 'International Tables Vol C Tables 4.2.6.8 and 6.1.1.4'

_symmetry_cell_setting            'Orthorhombic'
_symmetry_space_group_name_H-M    'C222(1) '
_symmetry_Int_Tables_number       20

loop_
 _symmetry_equiv_pos_as_xyz
 'x, y, z'
 '-x, -y, z+1/2'
 '-x, y, -z+1/2'
 'x, -y, -z'
 'x+1/2, y+1/2, z'
 '-x+1/2, -y+1/2, z+1/2'
 '-x+1/2, y+1/2, -z+1/2'
 'x+1/2, -y+1/2, -z'

_cell_length_a                    7.342(4)
_cell_length_b                    12.734(6)
_cell_length_c                    10.635(5)
_cell_angle_alpha                 90.00
_cell_angle_beta                  90.00
_cell_angle_gamma                 90.00
_cell_volume                      994.3(8)
_cell_formula_units_Z             4
_cell_measurement_temperature     293(2)
_cell_measurement_reflns_used     ?
_cell_measurement_theta_min       ?
_cell_measurement_theta_max       ?

_exptl_crystal_description        'plate'
_exptl_crystal_colour             'Blue'
_exptl_crystal_size_max           0.2
_exptl_crystal_size_mid           0.1
_exptl_crystal_size_min           0.05
_exptl_crystal_density_meas       'not measured'
_exptl_crystal_density_diffrn     5.789
_exptl_crystal_density_method     'not measured'
_exptl_crystal_F_000              1468
_exptl_absorpt_coefficient_mu     49.235
_exptl_absorpt_correction_type    'Multi-scan'
_exptl_absorpt_correction_T_min   0.481
_exptl_absorpt_correction_T_max   1.0000
_exptl_absorpt_process_details    ?

_exptl_special_details
;
```

```
 ?
;

_diffrn_ambient_temperature       293(2)
_diffrn_radiation_wavelength      0.71073
_diffrn_radiation_type            MoK\a
_diffrn_radiation_source          'fine-focus sealed tube'
_diffrn_radiation_monochromator   graphite
_diffrn_measurement_device_type   'Rigaku'
_diffrn_measurement_method        'CCD_Profile_fitting'
_diffrn_detector_area_resol_mean  5.8140
_diffrn_standards_number          ?
_diffrn_standards_interval_count  ?
_diffrn_standards_interval_time   ?
_diffrn_standards_decay_%         ?
_diffrn_reflns_number             2255
_diffrn_reflns_av_R_equivalents   0.1002
_diffrn_reflns_av_sigmaI/netI     0.1600
_diffrn_reflns_limit_h_min        -8
_diffrn_reflns_limit_h_max        8
_diffrn_reflns_limit_k_min        -15
_diffrn_reflns_limit_k_max        14
_diffrn_reflns_limit_l_min        -12
_diffrn_reflns_limit_l_max        12
_diffrn_reflns_theta_min          3.20
_diffrn_reflns_theta_max          25.48
_reflns_number_total              868
_reflns_number_gt                 431
_reflns_threshold_expression      >2sigma(I)

_computing_data_collection        'CrystalClear (Rigaku Corp.,2007)'
_computing_cell_refinement        'CrystalClear (Rigaku Corp.,2007)'
_computing_data_reduction         'CrystalClear (Rigaku Corp.,2007)'
_computing_structure_solution     'SHELXS-97 (Sheldrick, 1990)'
_computing_structure_refinement   'SHELXL-97 (Sheldrick, 1997)'
_computing_molecular_graphics     'DIAMOND (Brandenburg, 1999)'
_computing_publication_material   'Siemens SHELXTL^TM^ 5'

_refine_special_details
;
 Refinement of F^2^ against ALL reflections.  The weighted R-factor wR and
 goodness of fit S are based on F^2^, conventional R-factors R are based
 on F, with F set to zero for negative F^2^. The threshold expression of
 F^2^ > 2sigma(F^2^) is used only for calculating R-factors(gt) etc. and is
 not relevant to the choice of reflections for refinement.  R-factors based
 on F^2^ are statistically about twice as large as those based on F, and R-
 factors based on ALL data will be even larger.
;

_refine_ls_structure_factor_coef  Fsqd
_refine_ls_matrix_type            full
_refine_ls_weighting_scheme       calc
_refine_ls_weighting_details
 'calc w=1/[\s^2^(Fo^2^)+(0.0100P)^2^+0.0000P] where P=(Fo^2^+2Fc^2^)/3'
_atom_sites_solution_primary      direct
_atom_sites_solution_secondary    difmap
_atom_sites_solution_hydrogens    ?
_refine_ls_hydrogen_treatment     ?
_refine_ls_extinction_method      SHELXL
_refine_ls_extinction_coef        0.00049(7)
_refine_ls_extinction_expression
 'Fc^*^=kFc[1+0.001xFc^2^\l^3^/sin(2\q)]^-1/4^'
_refine_ls_abs_structure_details
 'Flack H D (1983), Acta Cryst. A39, 876-881'
_refine_ls_abs_structure_Flack    0.0(11)
_refine_ls_number_reflns          868
_refine_ls_number_parameters      50
```

```
_refine_ls_number_restraints      0
_refine_ls_R_factor_all           0.1232
_refine_ls_R_factor_gt            0.0742
_refine_ls_wR_factor_ref          0.1395
_refine_ls_wR_factor_gt           0.1171
_refine_ls_goodness_of_fit_ref    0.940
_refine_ls_restrained_S_all       0.940
_refine_ls_shift/su_max           0.000
_refine_ls_shift/su_mean          0.000

loop_
 _atom_site_label
 _atom_site_type_symbol
 _atom_site_fract_x
 _atom_site_fract_y
 _atom_site_fract_z
 _atom_site_U_iso_or_equiv
 _atom_site_adp_type
 _atom_site_occupancy
 _atom_site_site_symmetry_order
 _atom_site_calc_flag
 _atom_site_refinement_flags_posn
 _atom_site_refinement_flags_adp
 _atom_site_refinement_flags_occupancy
 _atom_site_disorder_assembly
 _atom_site_disorder_group
Hg1 Hg 0.48313(9) 0.5000 0.5000 0.0648(3) Uani 1 2 d S T P . .
Hg2 Hg 0.23501(6) 0.24972(6) 0.49258(8) 0.05608(18) Uani 1 1 d . . . . .
Co1 Co 0.0000 0.5010(3) 0.7500 0.0506(9) Uani 1 2 d S T P . .
S1 S 0.9914(4) 0.1671(3) 0.5958(3) 0.0344(9) Uani 1 1 d . . . . .
Cl1 Cl 0.5314(7) 0.0000 0.5000 0.104(3) Uani 1 2 d S T P . .
Cl2 Cl 0.0000 0.3248(4) 0.2500 0.0430(15) Uani 1 2 d S T P . .
Cl3 Cl 0.2356(3) 0.0880(2) 0.2600(4) 0.0329(9) Uani 1 1 d . . . . .

loop_
 _atom_site_aniso_label
 _atom_site_aniso_U_11
 _atom_site_aniso_U_22
 _atom_site_aniso_U_33
 _atom_site_aniso_U_23
 _atom_site_aniso_U_13
 _atom_site_aniso_U_12
Hg1 0.0512(4) 0.0311(4) 0.1120(6) -0.0348(5) 0.000 0.000
Hg2 0.0317(2) 0.0486(3) 0.0880(4) -0.0007(5) 0.0053(4) -0.0124(2)
Co1 0.0097(8) 0.01(1) 0.137(2) 0.000 -0.001(1) 0.000
S1 0.0247(14) 0.0282(18) 0.0504(19) 0.0041(19) -0.0072(17) -0.0050(18)
Cl1 0.110(4) 0.047(4) 0.155(6) 0.001(6) 0.000 0.000
Cl2 0.032(2) 0.007(2) 0.096(3) 0.000 0.000(3) 0.000
Cl3 0.0255(15) 0.01(1) 0.066(2) -0.007(2) -0.0101(18) 0.0080(11)

_geom_special_details
;
 All esds (except the esd in the dihedral angle between two l.s. planes)
 are estimated using the full covariance matrix.  The cell esds are taken
 into account individually in the estimation of esds in distances, angles
 and torsion angles; correlations between esds in cell parameters are only
 used when they are defined by crystal symmetry.  An approximate (isotropic)
 treatment of cell esds is used for estimating esds involving l.s. planes.
;

loop_
 _geom_bond_atom_site_label_1
 _geom_bond_atom_site_label_2
 _geom_bond_distance
 _geom_bond_site_symmetry_2
 _geom_bond_publ_flag
Hg1 S1 2.360(4) 8_456 ?
```

Hg1 S1 2.360(4) 5_455 ?
Hg2 S1 2.348(3) 1_455 ?
Hg2 S1 2.355(3) 8_456 ?
Co1 Cl2 2.218(6) 2_565 ?
Co1 Cl3 2.251(3) 8_456 ?
Co1 Cl3 2.251(3) 6 ?
Co1 Cl1 2.6687(13) 6 ?
Co1 Cl1 2.6687(13) 5_455 ?
S1 Hg2 2.348(3) 1_655 ?
S1 Hg2 2.355(3) 8_556 ?
S1 Hg1 2.360(4) 5_545 ?
Cl1 Co1 2.6687(13) 6_554 ?
Cl1 Co1 2.6687(13) 5_545 ?
Cl2 Co1 2.218(6) 2_564 ?
Cl3 Co1 2.251(3) 6_554 ?

loop_
 _geom_angle_atom_site_label_1
 _geom_angle_atom_site_label_2
 _geom_angle_atom_site_label_3
 _geom_angle
 _geom_angle_site_symmetry_1
 _geom_angle_site_symmetry_3
 _geom_angle_publ_flag
S1 Hg1 S1 177.06(14) 8_456 5_455 ?
S1 Hg2 S1 175.53(7) 1_455 8_456 ?
Cl2 Co1 Cl3 120.26(11) 2_565 8_456 ?
Cl2 Co1 Cl3 120.26(11) 2_565 6 ?
Cl3 Co1 Cl3 119.5(2) 8_456 6 ?
Cl2 Co1 Cl1 90.28(8) 2_565 6 ?
Cl3 Co1 Cl1 88.29(15) 8_456 6 ?
Cl3 Co1 Cl1 91.42(15) 6 6 ?
Cl2 Co1 Cl1 90.28(8) 2_565 5_455 ?
Cl3 Co1 Cl1 91.42(15) 8_456 5_455 ?
Cl3 Co1 Cl1 88.29(15) 6 5_455 ?
Cl1 Co1 Cl1 179.44(16) 6 5_455 ?
Hg2 S1 Hg2 102.75(13) 1_655 8_556 ?
Hg2 S1 Hg1 102.82(12) 1_655 5_545 ?
Hg2 S1 Hg1 102.29(12) 8_556 5_545 ?
Co1 Cl1 Co1 170.1(2) 6_554 5_545 ?

_diffrn_measured_fraction_theta_max 0.971
_diffrn_reflns_theta_full 25.48
_diffrn_measured_fraction_theta_full 0.971
_refine_diff_density_max 2.699
_refine_diff_density_min -2.534
_refine_diff_density_rms 0.467
